# A Cross-Sectional Study Evaluating the Effects of Resistance Exercise on Inflammation and Neurotrophic Factors in Elderly Women with Obesity

**DOI:** 10.3390/jcm9030842

**Published:** 2020-03-20

**Authors:** Hee-Tae Roh, Su-Youn Cho, Wi-Young So

**Affiliations:** 1 Department of Physical Education, College of Arts and Physical Education, Dong-A University, Busan 49315, Korea; dau0409@dau.ac.kr; 2 Exercise Physiology Laboratory, Department of Physical Education, Yonsei University, Seoul 03722, Korea; 3 Sports and Health Care Major, College of Humanities and Arts, Korea National University of Transportation, Chungju-si 27469, Korea

**Keywords:** elderly women, inflammation, neurotrophic factors, obesity, resistance exercise

## Abstract

Purpose: Aging lowers brain functionality, and obesity accelerates this process. Resistance exercise can help reverse aging; however, studies examining how it affects brain function and body mass are limited. Thus, this study aimed to investigate the effect of resistance exercise on inflammation and neurotrophic factors in elderly women with obesity. Methods: Twenty-six elderly women with obesity were selected for this study and randomly assigned into a control group (CG, *n* = 13) and an experimental group (EG, *n* = 13). The EG performed resistance training thrice weekly for 12 weeks using elastic bands, while the CG did not exercise. Serum lipid profile (total cholesterol (TC), triglyceride (TG), low density lipoprotein cholesterol (LDL-C), high density lipoprotein cholesterol (HDL-C)) and nuclear factor Kappa B (NF-κB), interferon-gamma (IFN-γ), brain-derived neurotrophic factor (BDNF), vascular endothelial growth factor (VEGF), and eotaxin-1 levels were analyzed before and after the intervention. Body composition (soft lean mass (SLM), skeletal muscle mass (SMM), body fat mass (BFM), percent body fat (PBF), waist-hip-ratio (WHR), basal metabolic rates (BMR)) measurements and blood tests were performed. Results: Among the body composition variables, SLM, SMM, and BMR in the EG were significantly increased after intervention (*p* < 0.05). Serum lipid profile was not significantly different after intervention (*p* > 0.05). After intervention, the levels of NF-κB, IFN-γ, and eotaxin-1 were significantly lower and BDNF and VEGF were significantly higher in the EG than in the CG (*p* < 0.05 for all). Conclusions: These results imply that regular resistance training in elderly women with obesity can increase muscle mass, reduce inflammation, and stimulate neurotrophic factors.

## 1. Introduction

Obesity has become a global health concern, with over 500 million and 1.4 billion adults worldwide classified to have obesity and be overweight, respectively [1]. Moreover, it is well known as the most common metabolic disease and is associated with insulin resistance, type 2 diabetes, hypertension, and cardiovascular disease [1,2].

Recently, obesity was reported to increase the incidence of dementia, cognitive function disorder, and Alzheimer’s disease, suggesting that it could negatively affect the structural and functional changes in the brain [1,2,3,4,5]. In particular, Geha et al. have shown that decreased functioning of the prefrontal cortex was observed in individuals with obesity [4], and other studies have demonstrated that obesity negatively affected the integrity of neural structures, including the gray and white matter of the brain [1,5].

In a study on 1289 adults whose mean cortical thickness was measured by MRI, Caunca et al. reported that obesity can accelerate the aging of brain cells, showing that the increases in body mass index (BMI) and waist circumference were related to gray matter atrophy [5]. Furthermore, it was shown that obesity induces brain atrophy and neurodegenerative diseases such as cognitive impairment and Alzheimer’s disease. In addition, chronic inflammation due to obesity is also assumed to cause neuroinflammation in the brain [6,7]. Likewise, obesity can have negative effects on the expression of neurotrophic factors (NTFs), such as the brain-derived neurotrophic factor (BDNF) and vascular endothelial growth factor (VEGF), which are involved in neurogenesis [8,9]. Namely, the NTFs play a role in neuronal growth, survival, and differentiation; they further regulate the release of neurotransmitters and plasticity of new synapses, while obesity induces the downregulation of NTF expression [9,10,11]. Sandrini et al. reported that the decrease in BDNF expression was associated with obesity in both human and animal models [12], which was supported by other studies showing that subjects with obesity and overweight had significantly lower peripheral BDNF and VEGF levels than normal individuals [13,14].

Alternatively, regular exercise effectively prevents the adverse effects of obesity and reduces it; therefore, it is an essential component of the management plan for various chronic diseases [15]. Aerobic training has anti-inflammatory effects that relieve chronic inflammation caused by obesity [16,17]. Additionally, it can promote brain function by inducing neurogenesis, and increasing neural circuitry and synaptic plasticity. A study also showed that increased NTFs mainly cause these beneficial effects due to exercise [18]. However, most studies on NTFs and neurogenesis are restricted to aerobic exercise only.

Resistance exercise is effective for inducing skeletal muscle hypertrophy and activating carbohydrate metabolism. Especially, it is recommended for elderly patients with low muscular strength due to skeletal muscle atrophy caused by aging. Skeletal muscle hypertrophy induces astrocyte activation and protein synthesis, thereby counteracting the negative effects caused by aging [19,20]. Additionally, resistance exercise was reported to stimulate lipolysis of adipose tissue in obesity patients [21,22], and free-living physical activity was shown to positively impact the body composition and resting metabolic rate by increasing the fat-free mass and daily energy expenditure in elderly people [22,23]. However, how resistance exercise affects brain function in elderly individuals with obesity is unclear. Thus, this study aimed to investigate the effect of resistance exercise on inflammation and neurotrophic factors in elderly women with obesity.

## 2. Methods

### 2.1. Participants

The prospective number of subjects was calculated as 24 for an effect size of 0.40, an α value of 0.05, and a desired statistical power (1-β) of 0.95, but 26 subjects were selected considering dropout. The participants were 26 elderly women who fully understood the purpose and significance of this study and voluntarily applied for it in the local community of Busan, Korea. The women were aged 70.58 ± 6.22 years with body fat percentage of 36.07 ± 2.51% (mean ± standard deviation (SD)). They were randomly assigned to the control group (CG, *n* = 13), which did not undergo any treatment for 12 weeks, and the experimental group (EG, *n* = 13), which underwent the resistance exercise program. All participants provided informed consent, including the consent to discontinue. The study protocol was approved by the ethics committee of the National Research Foundation of Korea (NRF-2017R1C1B5017956), and the study conformed to the standards set by the latest revision of the Declaration of Helsinki.

The selection criteria were as follows: (1) age above 60 years; (2) obesity with >32% body fat; (3) lack of any regular exercise; (4) absence of any musculoskeletal, neurological, or psychiatric disease. The exclusion criteria were as follows: (1) current or former smoking; (2) use of diet-related medications; (3) fasting glucose level ≥126 mg/dL (diabetes patient); (4) respiratory and/or cardiovascular disorders; (5) orthopedic disorders, which would negatively affect exercise performance; and (6) occupation that requires moderate physical activity. The characteristics of the participants at baseline are reported in Table 1, and all variables show no significant differences between groups (*p* > 0.05).

### 2.2. Research Procedure

The procedures in the study were subject selection, basic test, pre-test, and post-test before and after the 12-week intervention. In the basic test, a questionnaire was used to evaluate the existence of any disease or disability. In addition, excessive calorie intake and drinking were restricted, and excessive physical activity that could rapidly increase calorie consumption was inhibited during the experiment period based on nutrient consumption record covering seven days (including the weekend) letting them record all foods in a day using a 24-h retrospective method. Participation in exercise programs that could affect the study variables was also examined. In the pre-test, body composition was evaluated using the body composition analyzer Inbody 720 (Biospace, Seoul, Korea) through bioelectrical impedance analysis. Subsequently, blood samples were collected for evaluating the serum glucose level, lipid profile, inflammation markers, and neurotrophic factors. Further, the EG participated in the 12-week resistance exercise program, and the CG participants maintained their daily life without undergoing any intervention. In the post-intervention, the body composition analysis and blood tests were repeated to evaluate the post-interventional changes in EG participants.

### 2.3. Blood Collection and Analysis

For every participant, 10 mL of blood was drawn from the antecubital vein using a 22-gauge needle and a serum separator tube under resting conditions after 8 h of fasting. The blood was centrifugated at 3000 rpm for 15 min to separate the serum, which was then stored at −80 °C until the analysis of serum fasting glucose and lipid profile: total cholesterol (TC), triglyceride (TG), low-density lipoprotein cholesterol (LDL-C), high-density lipoprotein cholesterol (HDL-C). The following inflammation markers were evaluated: nuclear factor Kappa B (NF-κB) and interferon-gamma (IFN-γ); the following neurotrophic factors were evaluated: brain-derived neurotrophic factor (BDNF), vascular endothelial growth factor (VEGF), and eotaxin-1. The serum glucose level was determined by the hexokinase method using Roche Cobas 8000 modular analyzer and Glucose HK Gen.3 Kit (Roche Diagnostics GmbH, Mannheim, Germany). The serum TC (CHOL2, Roche Diagnostics GmbH, Mannheim, Germany), TG (TRIGL, Roche Diagnostics GmbH, Mannheim, Germany), and HDL-C (HDL-Cholesterol Gen. 4, Roche Diagnostics GmbH, Mannheim, Germany) levels were analyzed using enzymatic and colorimetric methods, and the LDL-C level was calculated using the Friedewald formula (LDL-C + TC − (HDL-C + TG/5)) [24]. In detail, 300 μL of enzyme solution was added to 2 μL of serum and stirred. TC and HDL-C and TG were allowed to react at 37 °C for 5 and 10 min, respectively, to develop colored products. Absorbance of TC and HDL-C was measured at 500 nm and of TG was measured at 550 nm using Roche Cobas 8000 modular analyzer (Roche, Germany). For analyzing the serum NF-κB, IFN-γ, BDNF, VEGF, and eotaxin-1 levels, we used the Human NF-κB Kit (CSB-E12107h, CUSABIO, Wuhan, China, datasheet available at https://www.cusabio.com/uploadfile/Ins/2018-01-09/CSB-E12107h.pdf), Human IFN-γ Kit (DY285B-05, R&D Systems, Minneapolis, MN, USA, datasheet available at https://resources.rndsystems.com/pdfs/datasheets/dy285b.pdf), Human BDNF Kit (DY248, R&D Systems, Minneapolis, MN, USA, datasheet available at https://resources.rndsystems.com/pdfs/datasheets/dy248.pdf), Human VEGF Kit (DY293B-05, R&D Systems, Minneapolis, MN, USA, datasheet available at https://resources.rndsystems.com/pdfs/datasheets/dy293b.pdf), and the Human CCL11/Eotaxin Kit (DY320, R&D Systems, Minneapolis, MN, USA, datasheet available at https://resources.rndsystems.com/pdfs/datasheets/dy320.pdf), respectively. During the calculations, the absorbance values were quantified at 450 nm using a sandwich enzyme-linked immunosorbent assay (ELISA) reader (Tecan Sunrise, TECAN GmbH, Salzburg, Austria).

### 2.4. Resistance Training Intervention

The EG patients underwent the resistance training thrice weekly for 12 weeks using elastic bands (Spoband 25, Spoband Co., Seoul, Korea). Every session consisted of 40-min main exercise, 10-min stretching for warm-up, and 10-min stretching to cool-down, for a total of 60 min. This exercise had a 10–14 intensity on the rating of perceived exertion (RPE) scale and consisted of 13 motions of resistance band exercises (alternate arm and leg lift, Charlie Chaplin, clam, curl up, the hundred, rocket jumps, rolling like a ball, leg raise, shoulder bridge, side knee open stretches, side double leg lift, Swan, and Swimming).

### 2.5. Statistical Analysis

For data analysis in this study, mean and standard deviation of all dependent variables were calculated using SPSS version 25.0 for Windows (IBM Corp., Armonk, NY, USA). Two-way repeated measured analysis of variance (ANOVA) was conducted to examine the differences in each dependent variable between the groups. An independent *t*-test and a dependent *t*-test were conducted for analyzing statistically significant associations, and the level of statistical significance was set at 0.05.

## 3. Results

### 3.1. Change in the Body Composition

The body composition changes before and after the intervention in the CG and EG are shown in Table 2. From the two-way repeated measures ANOVA that analyzed body composition, significant interactions of soft lean mass (F = 12.726, *p* = 0.002), skeletal muscle mass (F = 13.861, *p =* 0.001), and basal metabolic rate (F = 12.599, *p* = 0.002) were observed. In the post-hoc test, the CG showed significantly lower soft lean mass, skeletal muscle mass, and basal metabolic rates after the intervention than before the intervention (*p* < 0.05). Conversely, the EG showed significantly increased soft lean mass, skeletal muscle mass, and basal metabolic rates after the intervention (*p* < 0.05). However, no significant differences were observed in the weight, BMI, body fat mass, body fat %, and waist-hip ratio (*p* > 0.05).

### 3.2. Change in Fasting Glucose Levels and Lipid Profile

The fasting glucose level and lipid profile data of the CG and EG before and after intervention are shown in Table 3. On two-way repeated measures ANOVA for serum fasting glucose and lipid profile, significant associations between intervention time and groups were observed for fasting glucose (F = 5.970, *p* = 0.022) levels. The post-hoc test showed that the EG had significantly lower fasting glucose levels after the intervention (*p* < 0.05). However, no significant differences were observed in the TC, TG, LDL-C, and HDL-C levels (*p* > 0.05).

### 3.3. Change in Serum Inflammation Markers

The serum inflammation marker data of the CG and EG before and after intervention are shown in Table 4. The two-way repeated measures ANOVA showed significant association between the intervention time and groups in NF-κB (F = 6.084, *p* = 0.021) and IFN-γ (F = 6.764, *p* = 0.016) levels. The post-hoc test showed that the EG had significantly lower NF-κB after the intervention (*p* < 0.05). Furthermore, the EG showed significantly lower IFN-γ levels after the intervention than the CG (*p* < 0.05).

### 3.4. Change in Serum Neurotrophic Factors

The data of serum neurotrophic factors before and after intervention in the CG and EG are shown in Table 5. From the two-way repeated measures ANOVA for serum neurotrophic factors, significant associations between intervention time and groups were observed for BDNF (F = 28.496, *p* < 0.001), VEGF (F = 11.767, *p* = 0.002), and eotaxin-1 (F = 11.341, *p* = 0.003) levels. From the post-hoc test, EG showed significantly higher BDNF and VEGF levels (*p* < 0.05) but significantly lower eotaxin-1 levels (*p* < 0.05) after the intervention. Furthermore, the EG showed significantly higher BDNF levels after the intervention than the CG (*p* < 0.05).

## 4. Discussion

Aging is among the factors that decrease strength and function of muscles. The decrease in skeletal muscle mass (atrophy) begins at the age of 30–39 years, and muscle mass can reduce to approximately 10% when women are aged between 50 and 59 years. Muscle strength decreases at the rate of 2–4% every year in the age range of 50–60 years [25]. The mechanism causing the loss of muscle mass due to aging is not clear yet; however, suggested mechanisms include the decline of insulin sensitivity in skeletal muscle, decrease of muscle protein quantity, mitochondrial dysfunction, and disability of regenerative capacity. In particular, the increased body fat due to obesity is reported to exacerbate various metabolic problems in skeletal muscles.

Nevertheless, participating in physical activities including exercise is essential for healthy aging since it alleviates metabolic problems of skeletal muscles caused by aging and obesity. In particular, resistance exercises were proven as an effective way to increase muscle mass and strength [19,25]. Patients in our study also showed a significant increase in soft lean mass and skeletal muscle mass after the 12-week resistance exercise. This is consistent with the results of previous studies, where elastic band exercise training was effective in increasing the muscle mass in elderly women with aging-induced sarcopenic obesity [26,27]. This suggests that resistance exercise using elastic bands can be effective for improving body composition by inducing muscle mass increase in elderly women with obesity. Liao et al. reported a significant increase in fat-free mass in 46 elderly women with obesity and sarcopenia by a 12-week elastic resistance exercise regime using TheraBand [26]. Moreover, we ascertain that the basal metabolic rate significantly increased after the 12-week resistance exercise by the increase of muscle mass. Petridou et al. reported that the resting metabolic rate can be raised by increasing the fat-free mass through resistance exercises [22]. A previous study noted that the resting metabolic rate is highly correlated with the body cell mass or lean body mass, which supports this theory [28]. In addition, the fasting glucose level significantly decreased after 12 weeks of resistance training in this study. This result is in agreement with those shown in an advanced study that reported positive improvement in glycemic control through resistance exercise training [29,30], and it is assumed that the increase of muscle mass like increase of basal metabolic rate in this study would mainly work. Bweir et al. reported that resistance exercise training can be more effective in reducing glycated hemoglobin (HbA1c) levels than aerobic training in subjects with inactive type 2 diabetes (mean age 53.5 years) [29]. The study by Baldi and Snowling addressed the effect of 10 weeks of resistance training on type 2 diabetes patients with obesity and found significant reduction in fasting glucose and HbA1c levels, with a 3.5%-increase in fat-free mass despite no significant difference in fat mass; this suggested that fasting glucose and HbA1c levels are negatively correlated with fat-free mass [30]. However, there were no significant effects on BMI, percent body fat, waist-hip ratio, and lipid profile (TC, TG, LDL-C, HDL-C), which are the parameters for obesity measurements in our study. These results suggest that 12-week resistance exercise did not induce sufficient energy expenditure and lipolysis, which is necessary to reduce obesity. Previous studies noted that resistance training was insufficient to independently reduce obesity since even though it can stimulate adipose tissue lipolysis similar to that seen with endurance exercises in persons with obesity, resistance exercises include long resting periods that result in lower energy expenditure, as compared to endurance exercises; hence, fatty acids from lipolysis are not oxidized, but re-esterified to triacylglycerols [22,31]. However, a study by Gómez-Tomás et al. showed a significant decrease in waist circumference, TC, and LDL-C after 1 year of elastic band resistance exercise intervention [32]. Therefore, future studies should be conducted to examine this result in the context of a longer period of exercise.

Low-grade inflammation caused by obesity contributes to the occurrence of metabolic syndrome. Recently, various parts of the brain such as the hypothalamus, hippocampus, cerebellum, cerebral cortex, brainstem, and amygdala showed induced neuroinflammation in rodent obesity models [33]. The markers of neuroinflammation in the brain tissues are proinflammatory cytokines/chemokines such as interleukin (IL)-1β, IL-6, tumor necrosis factor-alpha, NF-κB, and IFN-γ, and the activation of microglia (microgliosis) and astrocytes (astrogliosis) [33,34]. Our study evaluated the changes in inflammation markers after resistance exercise by analyzing serum NF-κB and IFN-γ levels. The results of this study showed that NF-κB levels were significantly decreased after resistance exercise in the EG, and this group showed significantly lower IFN-γ levels than the CG. These results are congruent with the results of previous studies that reported a significant decrease in NF-κB and IFN-γ levels after exercise training in humans with metabolic syndrome [35,36,37] and those of studies showing that resistance exercise can alleviate neuroinflammation in rodent models [38]. Balducci et al. reported that anti-inflammatory effects of exercise training depended on exercise modalities, regardless of weight loss because serum IFN-γ levels of subjects with metabolic syndrome were significantly decreased when aerobic exercises were combined with anaerobic exercises rather than when only aerobic exercises were performed by patients with metabolic syndrome [36]. Moreover, Liu et al. reported that neuroinflammation in the frontal cortex and hippocampus of mice was improved after resistance training by climbing a ladder [37]. As already mentioned, the EG showed significant increases in soft lean mass, skeletal muscle mass, and basal metabolic rate. In contrast, the CG showed significant decreases in soft lean mass, skeletal muscle mass, and basal metabolic rate. An advanced epidemiologic study about sarcopenia in elderly people suggested that physical inactivity and chronic body inflammation are important reasons for the decrease in muscle mass in elderly people [39]; another advanced study showing that an increase in the secretion of inflammatory adipocytokines due to obesity promotes the catabolism of muscle [40]. Considering these findings, it is assumed that continuous chronic inflammation in the CG caused a significant decrease in soft lean mass and skeletal muscle mass, followed by a decrease in basal metabolic rate.

Regular exercise training can induce structural and functional improvements in the brain [41]. This beneficial effect occurs because of the increase in expression of NTFs, such as BDNF and VEGF, that induce the growth and survival of neurons [42], and decreases the expression of eotaxin-1, a neurogenesis inhibitor, eventually leading to a positive effect because of the exercise [43]. This study examined the change in neurotrophic factors expression following resistance exercise by analyzing serum BDNF, VEGF, and eotaxin-1 levels. In our study, after resistance exercise, BDNF and VEGF levels were significantly increased but eotaxin-1 levels were significantly decreased. These results are consistent with the results of previous studies, which reported that exercise significantly increased circulating BDNF and VEGF levels in subjects with obesity [11,44,45] and significantly decreased eotaxin-1 levels [43,46], suggesting that resistance exercises can be effective for ameliorating neurogenesis by increasing NTF expression and reducing eotaxin-1 levels similar to that by endurance exercises.

Resistance exercise that requires lower cardiorespiratory fitness than aerobic exercise has a beneficial effect on metabolic disease patients who are not able to perform aerobic exercises for a long time [47]. We found that resistance training alleviates the inflammation level and can be effective for the up-regulation of neurotrophic factors, even though it was not effective in alleviating the obesity rate in elderly women with obesity. However, this study has several limitations, and future research is required to address the questions raised in this study. First, because the participants were recruited from a local community center at Busan, Korea, they did not accurately represent the entire worldwide population of elderly women with obesity. Second, the sample size in this study was small, and only female subjects were enrolled; hence, the results may not be generalizable; future studies should examine sex-based differences by including male subjects, considering the finding that there could be sex-specific effects of resistance exercise on the change in circulating BDNF levels in elderly people [48]. Third, exercise can affect calorie intake, but daily calorie intake and the components of nutrients were not analyzed in this study. Future studies should examine these factors using computer-aided nutrition programs. Fourth, it only examined peripheral circulating markers, such as serum NF-κB, IFN-γ, BDNF, and VEGF, making it difficult to conclude that resistance exercise had positive effect on neuroinflammation and neurogenesis at the central nervous system level; therefore, future studies should include noninvasive methods, such as fMRI, that can examine brain function and structural changes in order to evaluate brain function with higher precision.

## 5. Conclusions

In conclusion, resistance training using elastic bands could not significantly improve obesity and lipid profiles in elderly women with obesity. However, our results suggest that it can be effective for stimulating neurotrophic factors, reducing inflammation, and increasing muscle mass independently.

## Figures and Tables

**Table 1:** The characteristics of the participants at baseline.

Group	CG (*n* = 13)	EG (*n* = 13)	*p*
**Variables**			
Age (years)	70.23 ± 6.06	70.92 ± 6.60	0.783
Height (cm)	153.23 ± 4.75	153.58 ± 5.06	0.859
Weight (kg)	60.45 ± 6.07	57.72 ± 4.97	0.222
BMI (kg/m^2^)	25.72 ± 2.32	24.67 ± 1.55	0.186
SLM (kg)	35.36 ± 3.18	34.57 ± 2.96	0.519
SMM (kg)	20.12 ± 1.99	19.70 ± 1.82	0.583
BFM (kg)	22.82 ± 3.76	20.85 ± 2.62	0.136
PBF (%)	37.60 ± 3.40	36.07 ± 2.51	0.203
WHR	0.91 ± 0.06	0.89 ± 0.03	0.322
BMR (kcal)	1182.69 ± 72.15	1166.31 ± 67.26	0.555
Glucose (mg/dL)	103.92 ± 11.63	108.23 ± 12.32	0.368
TC (mg/dL)	189.46 ± 55.76	183.92 ± 43.65	0.780
TG (mg/dL)	139.00 ± 49.64	166.85 ± 98.44	0.372
LDL-C (mg/dL)	108.82 ± 56.18	100.48 ± 35.85	0.656
HDL-C (mg/dL)	52.85 ± 16.80	50.08 ± 8.55	0.601
NF-κB (ng/mL)	4.66 ± 1.61	4.51 ± 1.36	0.798
IFN-γ (pg/mL)	10.12 ± 3.57	9.96 ± 3.82	0.913
BDNF (ng/mL)	1.43 ± 0.16	1.55 ± 0.26	0.197
VEGF (pg/mL)	56.88 ± 11.08	58.15 ± 11.08	0.773
Eotaxin-1 (pg/mL)	8.86 ± 2.92	9.63 ± 2.63	0.485

Data are presented as mean ± standard deviation. CG, control group; EG, experimental group; BMI, body mass index; SLM, soft lean mass; SMM, skeletal muscle mass; BFM, body fat mass; PBF, percent body fat; WHR, waist-hip ratio; BMR, basal metabolic rate; TC, total cholesterol; TG, triglyceride; LDL-C, low-density lipoprotein cholesterol; HDL-C, high-density lipoprotein cholesterol; NF-κB, nuclear factor Kappa B; IFN-γ, interferon-gamma; BDNF, brain-derived neurotrophic factor; VEGF, vascular endothelial growth factor; *p*-value as determined using the independent *t*-test for each of the two groups at baseline.

**Table 2:** The changes in body composition.

Group	CG (*n* = 13)		EG (*n* = 13)		Time × Group Interaction	
Variables	Baseline	12-Week	Baseline	12-Week	F	*p*
Weight (kg)	60.45 ± 6.07	59.25 ± 5.66	57.72 ± 4.97	57.23 ± 5.10	3.872	0.061
CV	0.10	0.10	0.09	0.09		
BMI (kg/m^2^)	25.72 ± 2.32	25.29 ± 2.41	24.67 ± 1.55	24.38 ± 1.55	0.692	0.414
CV	0.09	0.10	0.06	0.06		
SLM (kg)	35.36 ± 3.18	34.81 ± 3.13 †	34.57 ± 2.96	35.17 ± 2.96 #	12.726	0.002 *
CV	0.09	0.09	0.09	0.09		
SMM (kg)	20.12 ± 1.99	19.77 ± 1.94 †	19.70 ± 1.82	20.07 ± 1.99 #	13.861	0.001 *
CV	0.10	0.10	0.09	0.10		
BFM (kg)	22.82 ± 3.76	22.21 ± 3.67	20.85 ± 2.62	19.79 ± 2.78	0.942	0.342
CV	0.16	0.17	0.13	0.14		
PBF (%)	37.60 ± 3.40	37.37 ± 3.82	36.07 ± 2.51	34.52 ± 3.08	24.000	0.057
CV	0.09	0.10	0.07	0.09		
WHR	0.91 ± 0.06	0.90 ± 0.05	0.89 ± 0.03	0.90 ± 0.03	3.450	0.076
CV	0.07	0.06	0.04	0.03		
BMR (kcal)	1182.69 ± 72.15	1169.69 ± 69.97 †	1166.31 ± 67.26	1178.77 ± 74.72 #	12.599	0.002 *
CV	0.06	0.06	0.06	0.06		

Data are presented as mean ± standard deviation. CG, control group; EG, experimental group; CV, coefficient of variation; BMI, body mass index; SLM, soft lean mass; SMM, skeletal muscle mass; BFM, body fat mass; PBF, percent body fat; WHR, waist-hip ratio; BMR, basal metabolic rate; † Compared with baseline values within CG (*p* < 0.05); # Compared with baseline values within EG (*p* < 0.05); * *p* < 0.05.

**Table 3:** The changes in the lipid profile.

Group	CG (*n* = 13)		EG (*n* = 13)		Time × Group Interaction	
Variables	Baseline	12-Week	Baseline	12-Week	F	*p*
Glucose (mg/dL)	103.92 ± 11.63	106.00 ± 20.02	108.23 ± 12.32	97.77 ± 13.78 #	5.970	0.022 *
CV	0.11	0.19	0.11	0.14		
TC (mg/dL)	189.46 ± 55.76	185.38 ± 45.64	183.92 ± 43.65	194.54 ± 43.84	1.177	0.634
CV	0.29	0.25	0.24	0.23		
TG (mg/dL)	139.00 ± 49.64	102.85 ± 30.03	166.85 ± 98.44	121.23 ± 58.99	0.088	0.769
CV	0.36	0.29	0.59	0.49		
LDL-C (mg/dL)	108.82 ± 56.18	109.43 ± 45.31	100.48 ± 35.85	118.06 ± 40.45	2.333	0.140
CV	0.52	0.41	0.36	0.34		
HDL-C (mg/dL)	52.85 ± 16.80	55.38 ± 13.89	50.08 ± 8.55	52.23 ± 12.25	0.010	0.920
CV	0.32	0.25	0.17	0.23		

Data are presented as mean ± standard deviation. CG, control group; EG, experimental group; CV, coefficient of variation; TC, total cholesterol; TG, triglyceride; LDL-C, low-density lipoprotein cholesterol; HDL-C, high-density lipoprotein cholesterol; # Compared with baseline values within EG (*p* < 0.05); * *p* < 0.05.

**Table 4:** The changes in the serum inflammation markers.

Group	CG (*n* = 13)		EG (*n* = 13)		Time × Group Interaction	
Variables	Baseline	12-Week	Baseline	12-Week	F	*p*
NF-κB (ng/mL)	4.66 ± 1.61	4.78 ± 1.68	4.51 ± 1.36	4.23 ± 1.24 #	6.084	0.021 *
CV	0.35	0.35	0.30	0.29		
IFN-γ (pg/mL)	10.12 ± 3.57	11.20 ± 4.15	9.96 ± 3.82	7.85 ± 2.80 ∫	6.764	0.016 *
CV	0.35	0.37	0.38	0.36		

Data are presented as mean ± standard deviation. CG, control group; EG, experimental group; CV, coefficient of variation; NF-κB, nuclear factor Kappa B; IFN-γ, interferon-gamma; # Compared with baseline values within EG (*p* < 0.05); ∫ Compared with corresponding values in CG (*p* < 0.05); * *p* < 0.05.

**Table 5:** The changes in the serum neurotrophic factors.

Group	CG (*n* = 13)		EG (*n* = 13)		Time × Group Interaction	
Variables	Baseline	12-Week	Baseline	12-Week	F	*p*
BDNF (ng/mL)	1.43 ± 0.16	1.32 ± 0.18	1.55 ± 0.26	1.61 ± 0.22 #,∫	28.496	<0.001 *
CV	0.11	0.14	0.17	0.14		
VEGF (pg/mL)	56.88 ± 11.08	55.67 ± 10.03	58.15 ± 11.08	62.64 ± 13.27 #	11.767	0.002 *
CV	0.19	0.18	0.19	0.21		
Eotaxin-1 (pg/mL)	8.86 ± 2.92	9.50 ± 3.03	9.63 ± 2.63	8.15 ± 3.15 #	11.341	0.003 *
CV	0.33	0.32	0.27	0.39		

Data are presented as mean ± standard deviation. CG, control group; EG, experimental group; CV, coefficient of variation; BDNF, brain-derived neurotrophic factor; VEGF, vascular endothelial growth factor; # Compared with baseline values within EG (*p* < 0.05); ∫ Compared with corresponding values in CG (*p* < 0.05); * *p* < 0.05.
